# Investigation of bio-based rigid polyurethane foams synthesized with lignin and castor oil

**DOI:** 10.1038/s41598-024-64318-8

**Published:** 2024-06-12

**Authors:** Hyeon Jeong Kim, Xuanjun Jin, Joon Weon Choi

**Affiliations:** 1 Graduate School of International Agricultural Technology, Seoul National University, Pyeongchang, 25354 Republic of Korea; 2 Institute of Green-Bio Science and Technology, Seoul National University, Pyeongchang, 25354 Republic of Korea

**Keywords:** Lignin, Castor oil, Polyurethane foam, Bio-based, Environmental sciences, Environmental chemistry

## Abstract

In this study, polyurethane (PU) foams were manufactured using kraft lignin and castor oil as bio-based polyols by replacing 5–20 wt% and 10–100 wt% of conventional polyol, respectively. To investigate the effects of unmodified bio-based polyols on PU foam production, reactivity and morphology within PU composites was analyzed as well as mechanical and thermal properties of the resulting foams. Bio-based PU foam production was carried out after characterizing the reagents used in the foaming process (including hydroxyl group content, molecular weight distribution, and viscosity). To compare the resulting bio-based PU foams, control foam were produced without any bio-based polyol under the same experimental conditions. For lignin-incorporated PU foams, two types, LPU and lpu, were manufactured with index ratio of 1.01 and 1.3, respectively. The compressive strength of LPU foams increased with lignin content from 5 wt% (LPU5: 147 kPa) to 20 wt% (LPU20: 207 kPa), although it remained lower than that of the control foam (PU0: 326 kPa). Similarly, the compressive strength of lpu foams was lower than that of the control foam (pu0: 441 kPa), with values of 164 kPa (lpu5), 163 kPa (lpu10), 167 kPa (lpu15), and 147 kPa (lpu20). At 10 wt% lignin content, both foams (LPU10 and lpu10) exhibited the smallest and most homogenous pore sizes and structures. For castor oil-incorporated PU foams with an index of 1.01, denoted as CPU, increasing castor oil content resulted in larger cell sizes and void fractions, transitioning to an open-cell structure and decreasing the compressive strength of the foams from 284 kPa (CPU10) to 23 kPa (CPU100). Fourier transform infrared (FT-IR) results indicated the formation of characteristic urethane linkages in PU foams and confirmed that bio-based polyols were less reactive with isocyanate compared to traditional polyol. Thermogravimetric analysis (TGA) showed that incorporating lignin and castor oil affected the thermal decomposition behavior. The thermal stability of lignin-incorporated PU foams improved as the lignin content increased with char yields increasing from 11.5 wt% (LPU5) to 15.8 wt% (LPU20) and from 12.4 wt% (lpu5) to 17.5 wt% (lpu20). Conversely, the addition of castor oil resulted in decreased thermal stability, with char yields decreasing from 10.6 wt% (CPU10) to 4.2 wt% (CPU100). This research provides a comprehensive understanding of PU foams incorporating unmodified biomass-derived polyols (lignin and castor oil), suggesting their potential for value-added utilization as bio-based products.

## Introduction

Polyurethane (PU) has become an integral part of daily life, providing convenience to humans through its use in applications such as coatings, elastomers, adhesives, and foams^1^. PU foams are widely used and can be flexible, semi-rigid, or rigid^2^. They dominate the PU market because of their use in residential and infrastructure construction, where they offer significant energy savings over other materials^3^. PU production typically involves a reaction between polyols and isocyanates, which are petroleum-derived materials^4^. Because of environmental concerns, bio-based PU is being developed; its market size was valued at USD 34.73 million in 2020, and it is expected to grow (Grand View Research, 2023). As alternatives to petroleum-based materials, biomass-derived materials such as lignin and vegetable oil are candidates that might serve as bio-based polyols to replace conventional polyols^5^.

**Figure 1:**
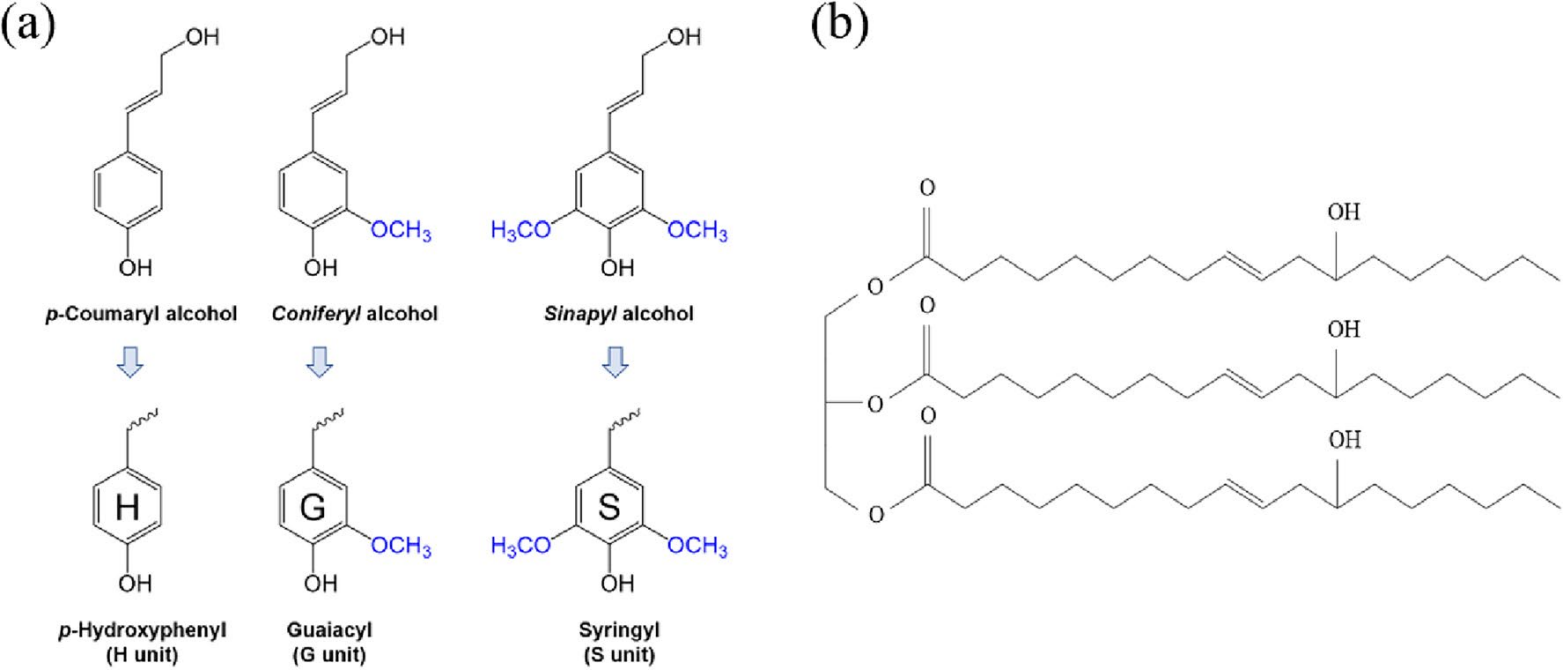
(**a**) Three lignin precursors (*p*-coumaryl, coniferyl, sinapyl alcohol) and their phenylpropane units, (**b**) Structural feature of castor oil.

Lignin (25–30%) is a major component of lignocellulosic biomass; the total amount of lignin in the biosphere exceeds 300 billion tons^6^. As shown in Fig. 1, lignin consists of *p*-hydroxyphenyl (H unit), guaiacyl (G unit), and syringyl (S unit) monomers that are derived from three types of monolignols (*p*-coumaryl, coniferyl, and sinapyl alcohol). Phenylpropane (C6C3) units (H, G, S units) are linked by several types of covalent bonds, including ether bonds (β-O-4, α-O-4, 4-O-5) and carbon–carbon bonds (β-β’, β-1’, β-5’, 5–5’)^7^. Most lignin is produced as a byproduct of the pulp and paper industry, and it is called “technical lignin” (or “industrial lignin”). Some parts of lignin can also be produced as a byproduct of biorefinery processes such as bioethanol and saccharification^8,9^. However, most lignin is used as an energy resource through combustion to generate electricity for the pulping process^10^. Even though its structure varies depending on the isolation processes and feedstock, lignin has received attention for its potential in diverse applications^11^. It can serve as a valuable feedstock in various fields (battery material, aromatic and flavoring compounds, dispersants, binders, flame retardants, antioxidants, PU, pharmaceuticals, drug delivery, etc.)^12,13^ because of its major functional groups: hydroxyl, methoxy, and carbonyl groups^14^. In this research, lignin, which contains hydroxyl functional groups, was used as a biopolyol to partially replace the traditional polyol in making PU foams^13^. Moreover. for the industrial production of lignin-derived chemicals and compounds, various network and interdisciplinary research has been conducted to explore the transformation of lignin into high-value products. Pulp companies are also showing interest in using lignin residue to create bio-based materials with added value, including binders, phenolic resins, composites, and batteries^6^.

Castor oil is a low-cost, abundantly available material extracted from the seed of castor oil plants. It is not edible, so its industrial use doesn’t contribute to the food availability crisis^15^. Castor oil has a triglyceride fatty acid structure that contains ricinoleic acid (about 90%), one double (C=C) bond, and one –OH group (secondary hydroxyl group) (Fig. 1). The hydroxyl group means that castor oil can be used as a polyol to synthesize cost-effective, bio-based PU^16^. Despite its many potential uses, castor oil has not yet been used in large industries, though it is being used in personal care and cosmetic products and therapeutics^17^. However, the growing interest in green and sustainable materials has brought attention to castor oil as a biomass-derived alternative to petroleum products^18–20^.

Research has been conducted to modify lignin and castor oil to improve their reactivity in PU production^21–23^; however, several of the proposed methods also have cost and toxicity issues^20,24^. In this study, the facile and low-cost production of PU foams was achieved by using unmodified lignin and castor oil in various ratios as partial substitutes for polyol. Relatively few studies have been conducted on bio-based PU foams using unmodified polyols compared to those using modified polyols^25–28^. Moreover, foundational and crucial information about the experimental process, particularly the foam manufacturing with characterization of bio-based polyol, has been more comprehensively detailed than in other bio-based PU foam studies. The objective of this study was to investigate the effects of biomass-derived polyols on the production of PU foams. Since unmodified lignin and castor oil can be expected to alter the properties of PU foams in a desired manner^25–28^, the resulting foams were subjected to thermal and mechanical analysis.

## Materials and methods

### Materials

The softwood kraft lignin (KL) used in this study was obtained through the Lignoboost process and generously provided by Domtar in the USA. To prepare the sample, the KL was finely powdered using a 500 µm mesh sieve. The polymeric Methylene Diphenyl Diisocyanate (pMDI) used, Lupranate M5S, with an NCO group content of 32%, was kindly supplied by BASF Korea. Polyethylene glycol 400 (PEG 400) and tetrahydrofuran (THF) were purchased from Daejung Science (Korea). Castor oil from SMGREEN (Korea) and silicone oil from Shin-Etsu (Japan) were used. Glycerol and dibutyltin dilaurate (DBTL) were purchased from Sigma-Aldrich (USA).

### Characterization of lignin

The ash content of the lignin samples was determined to find the inorganic weight percent. After pre-treatment of the crucibles and lids, they were weighed, and 500 mg of lignin was loaded into each crucible. Three crucibles were then placed in a furnace and heated to 550 °C for 5 h. After they were cool, the samples were transferred to a desiccator and weighed. The percentage of ash content was calculated as the difference between the weight of the ash and the original lignin sample.

An elemental analysis was performed using a US/CHNS-932 (LECO Corp., USA) to determine the carbon (C), nitrogen (N), hydrogen (H), and sulfur (S) content in the KL. The C, H, N, and S content were subjected to a high-temperature reaction with pure oxygen, leading to the production of CO_2_, H_2_O, N_2_, and SO_2_ gases, respectively. After purification, the gases were separated through a column. Quantifiable data for the C, H, N, and S content were obtained using a thermal conductivity detector. The oxygen content was calculated as the differences.

Fourier transform infrared (FT-IR) spectroscopy was performed to analyze the functional groups of lignin in the 4000 to 600 cm^−1^ range. Measurements were performed using 32 scans and a resolution of 8 on a Vertex-80 V/Hyperion2000 (BRUKER, Germany).

A liquid ^31^P nuclear magnetic resonance (NMR) analysis was performed to quantify the free hydroxyl groups in the KL. Before the NMR analysis, 50 ml of solvent solution were prepared by combining pyridine and deuterated chloroform (CDCl_3_) in a ratio of 1.6:1 (v/v). Then, to create the mixture solution, 100 mg of cyclohexanol (as an internal standard) and 90 mg of chromium (III) acetylacetonate (as a relaxation agent) were mixed with 25 ml of the prepared solvent solution. Subsequently, 15–20 mg of lignin were dissolved in the solvent solution (400 µL) and mixture solution (150 µL) and thoroughly stirred. For the phosphitylation reaction, 70 µL of 2-chloro-4,4,5,5-tetramethyl-1,3,2-dioxaphospholane reagent was added to the solution, and the mixture was shaken rapidly by hand. The resulting reaction mixture was then transferred to an NMR tube for analysis^29^. The ^31^P NMR spectra were acquired on a Bruker AVANCE 600 (Bruker, Germany) using a relaxation delay of 2 s and 256 scans. MestReNova^®^ software was used for the spectral analysis.

### The molecular weight distributions and viscosity of the polyols

A gel permeation chromatography analysis was conducted to investigate the molecular weight distributions of KL, castor oil, and PEG400. KL was dissolved in THF after acetylation, whereas the castor oil and PEG400 were directly dissolved in THF. Then each solvent mixture was filtered using a 0.2 µm syringe filter, and the molecular weight distribution was determined using a 1260 Infinity II LC System (Agilent Technologies Inc., USA) equipped with a PLgel 5 μm MIXED-C column (300 mm × 7.5 mm, Agilent Technologies Inc., USA). A molecular weight calibration curve was obtained using molecular polystyrene standards (Mp 266–66,000 Da, PSS Polymer Standards Service GmbH, Germany). The viscosity of castor oil and PEG400 was measured on an SV-100 viscometer (AND, Japan).

### Calculation of the amount of MDI required using the hydroxyl number

To determine the amount of MDI needed to achieve the desired property in a polyol reaction, various factors need consideration^30^: the target index, MDI equivalent weight (MDI eq.wt.), polyol equivalent weight (polyol eq.wt.), parts by weight (pbw), and presence of any water. The equivalent weight (eq.wt.) indicates the number of grams of a product needed to have one equivalent of reactive groups^31^.

By considering these parameters, the total weight of MDI required can be calculated using Eq. (1) ^27,32^.

1 $$\begin{aligned} {\text{Total weight of MDI required}} = \,& \left( {{\text{Index}}} \right)\left( {{\text{MDI eq}}.{\text{wt}}.} \right) \\ & \times { }\left( {\frac{polyol\, A \,pbw }{{polyol\, A\, eq.wt.}} + \frac{polyol\, B\, pbw }{{polyol\, B\, eq.wt.}}} \right. \\ & \left. { + \ldots + \frac{polyol\, N\, pbw }{{polyol\, N\, eq.wt.}} + \frac{pbw\, H2O}{{18/2}} } \right) \\ \end{aligned}$$

MDI eq.wt. = 4202/NCO (unit: g/eq).

polyol eq.wt. = 56,100/hydroxyl number (unit: g/eq).

The hydroxyl numbers of lignin, glycerol, PEG 400, and castor oil were measured following the procedures outlined in ASTM D4274-23 and ASTM E222. Initially, each sample was acetylated with a solution of acetic anhydride in pyridine. Each acetylated mixture was then placed in a glass bottle with a cap in a 105 °C oven for 2–3 h. Throughout this process, the solution was shaken every 20 min. Blank determination was concurrently conducted by omitting the sample from the acetylated mixture. Each hydroxyl number was calculated using Eq. (2).

2 $${\text{Hydroxyl number }}\left( {{\text{mg KOH}}/{\text{g}}} \right) \, = \frac{{\left( {{\text{A}} - {\text{B}}} \right){\text{ X Nt X}}56.1}}{{\text{W}}}{ }$$

A = KOH solution required for blank (mL); B = KOH solution required for sample (mL); Nt = solution at the temperature during analysis (meg/mL); 56.1 = molar mass of KOH (g/mol); W = sample used (g).

### Synthesis of bio-based PU foams using lignin or castor oil

**Figure 2:**
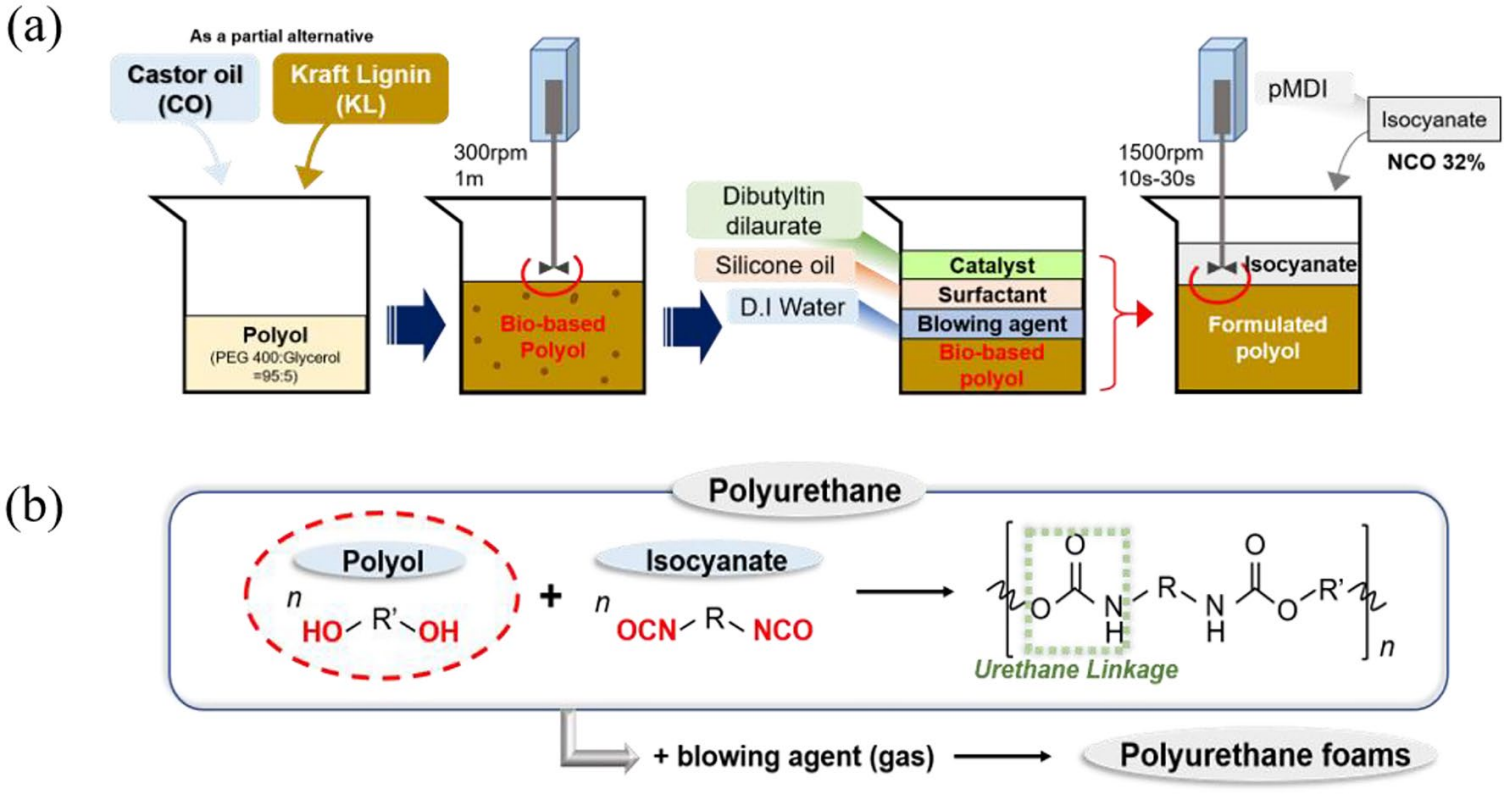
(**a**) Overall manufacture process of bio-based PU foam using one pot method, (**b**) Urethane linkage from the reaction between dialcohol and diisocyanate, and synthesis of polyurethane foams with the addition of blowing agent.

**Figure 3:**
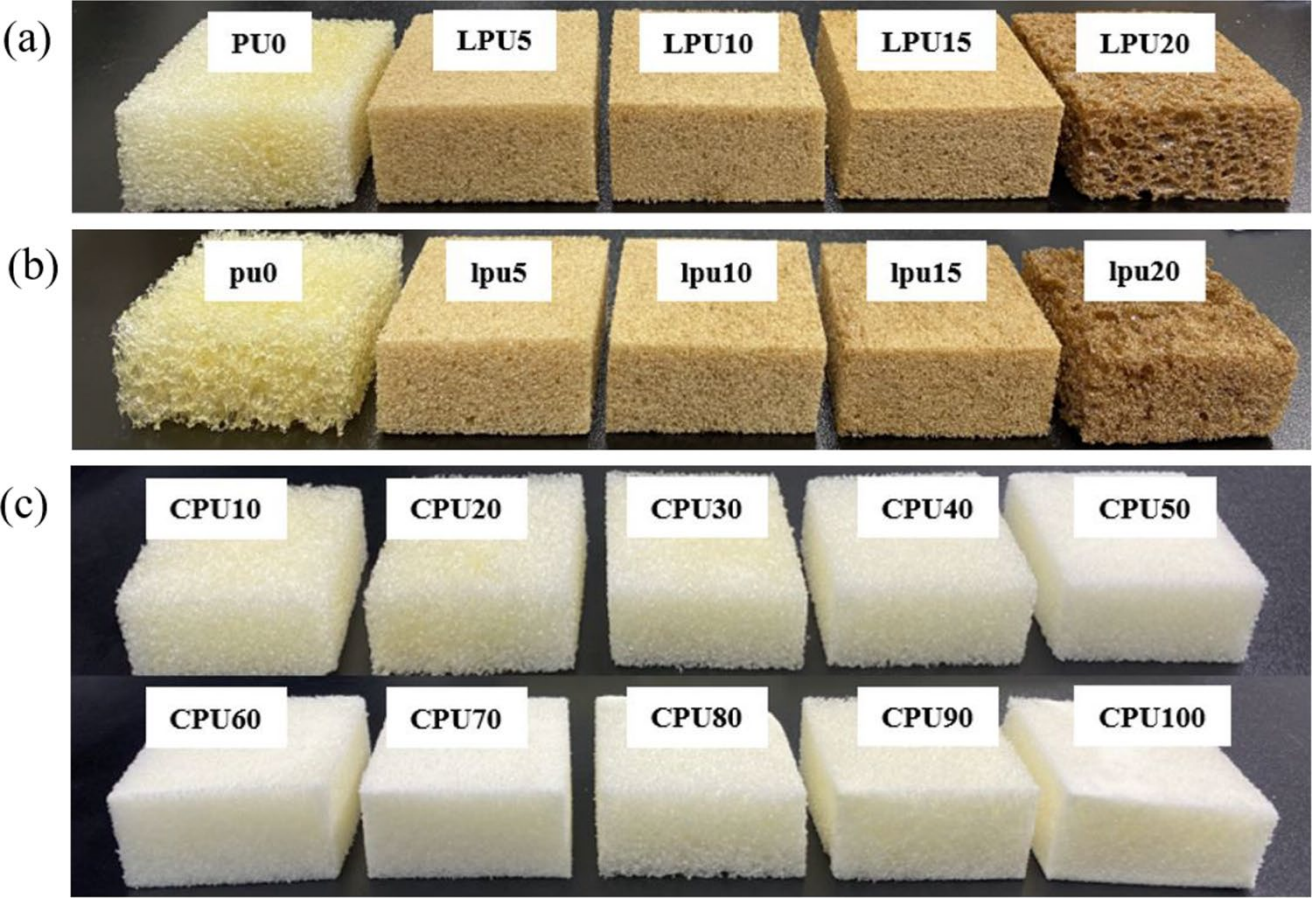
Bio-based polyurethane foams prepared by cutting into dimensions of 50 mm × 50 mm × 25 mm. (**a**) LPU with an index of 1.01, (**b**) lpu with an index of 1.3, (**c**) CPU with an index of 1.01.

The PU foam reaction mechanism and one-pot manufacture process are illustrated in Fig. 2. Initially, a bio-based polyol was prepared by physically combining PEG 400 and glycerol (95:5 w:w) and then adding the chosen bio-based material^26^. The resulting bio-based polyol was stirred with an overhead stirrer until homogeneity was achieved. The formulated polyol was then created by adding a catalyst (1 wt% of DBTL), surfactant (2 wt% of silicone oil), and blowing agent (2 wt% of water) into the bio-based polyol mixture^33^. Subsequently, the required amount of isocyanate, calculated by Eq. (1), was introduced into the formulated polyol and stirred vigorously at 1500 rpm for 10–30 s^27^. The formulations of the PU foams are detailed in Table 1. The resulting mixtures were allowed to cure at room temperature with exposure to the open air for a minimum of 24 h. After curing, each foam was prepared for analysis by cutting it into 50 mm × 50 mm × 25 mm pieces (Fig. 3).

**Table 1:** Formulations of bio-based polyurethane foams using lignin and castor oil as partial substitutes.

Index	Sample	A (wt%)						B (wt%)
		Castor oil	KL	PEG400 + Glycerol (95:5)	DBTL	Silicone oil	Water	pMDI
1.01	PU0		0	100	1	2	2	101
	LPU5		5	95				101
	LPU10		10	90				101
	LPU15		15	85				101
	LPU20		20	80				101
1.3	pu0		0	100				130
	lpu5		5	95				130
	lpu10		10	90				130
	lpu15		15	85				130
	lpu20		20	80				130
1.01	CPU10	10		90				101
	CPU20	20		80				101
	CPU30	30		70				101
	CPU40	40		60				101
	CPU50	50		50				101
	CPU60	60		40				101
	CPU70	70		30				101
	CPU80	80		20				101
	CPU90	90		10				101
	CPU100	100		0				101

To produce the lignin-based PU foams, lignin partially replaced the conventional polyol at 5 wt%, 10 wt%, 15 wt%, and 20 wt%. The resulting foams with different indexes, namely 1.01 and 1.3, are designated as LPU and lpu, respectively. For comparative purposes, control polyurethane foams with the different indexes but containing no bio-based materials are denoted as PU0 and pu0, respectively. To produce castor oil–based PU foams, castor oil replaced the conventional polyol in increments of 10 wt%, eventually reaching a 100 wt% substitution level. The resulting foams are characterized by their dependence on castor oil amount and designated as CPU.

### Characterization of bio-based PU foams

FT-IR spectra were analyzed to investigate the successful polyaddition reaction between polyol and Isocyanate. Compressive strength, the stress at 10% deformation, was measured according to ASTM D1621 to determine the mechanical properties of the PU foams. Each test specimen was 50 mm × 50 mm × 25 mm, and at least five samples were analyzed on a universal testing machine (34SC-1, Instron, USA) to obtain an average value. The test proceeded at the rate of 2.5 mm/min. The apparent density of the foams was determined in accordance with ASTM D1622. To obtain the weight/volume ratio, the length, width, and depth of each sample were measured using a digital caliper with a precision of 0.01 mm. Additionally, the samples were weighed on a digital balance with a precision of 0.1 mg. The density (kg/m^3^) was calculated based on measurements from at least five samples, each having the dimensions of 50 mm × 50 mm × 25 mm. The morphology of the foams was observed using a scanning electron microscope (SEM; TM3030 Plus, Hitachi, Japan). The thermal decomposition behavior of the bio-based PU foams was characterized using a thermogravimetric (TG) analysis on a TGA/DSC3 + (Mettler Toledo, Switzerland). The analysis was performed at a constant heating rate of 10 °C/min up to 800 °C in an inert N_2_ atmosphere with 50 mL/min of flow.

## Results and discussion

### Chemical composition of lignin

The chemical composition of lignin is presented in Table 2. Generally, lignin is composed of 60–65% carbon, 5–7% hydrogen, and 30–35% oxygen^34^. The lignin used in this study was composed of 61.2% carbon, 5.8% hydrogen, and 30.7% oxygen and contained no nitrogen. The ash content of the lignin was 2.3wt%. The inorganic compounds were investigated, and a small amount of sulfur (1.7 w%) was observed as a result of the kraft pulping process.

**Table 2:** Quantification of hydroxyl groups and chemical composition of kraft lignin.

Sample	Hydroxyl (mmol/g)						Elemental analysis (wt%)					Ash content (wt%)
	Phenolic			Carboxylic Acids	Aliphatic	Total (mmol/g)						
	H	G	S				C	H	N	O^a^	S	
KL	0.21	2.18	0.67	0.24	1.61	4.91	61.2	5.8	–	30.7	1.7	2.3 ± 0.03

^a^Calculated as the difference.

### Functional groups and hydroxyl groups of lignin

**Figure 4:**
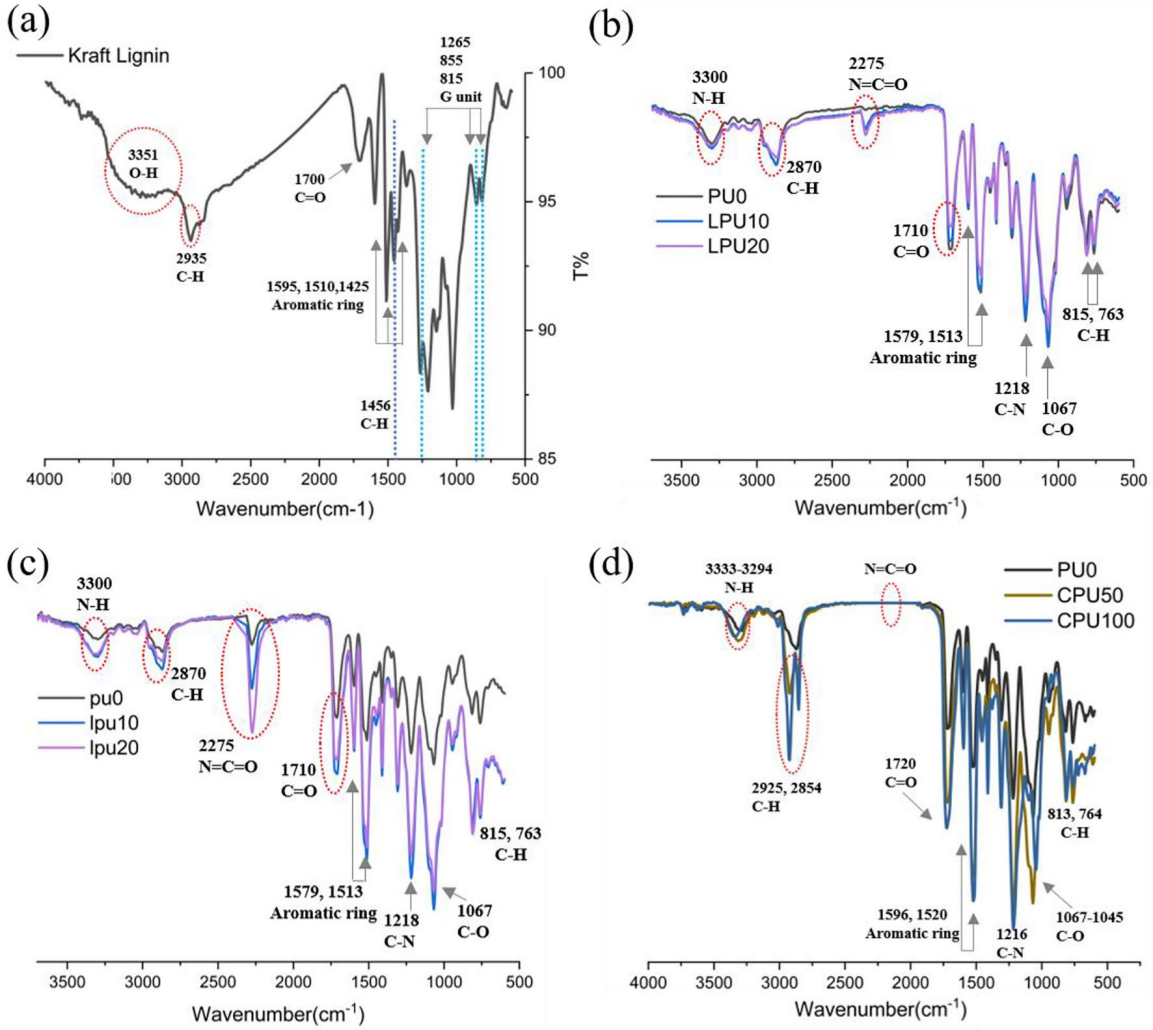
FTIR spectra of (**a**) kraft lignin, (**b**) LPU, (**c**) lpu and (**d**) CPU.

The FT-IR spectra of lignin are shown in Fig. 4a. An O–H stretching vibration from the hydroxyl groups (aromatic and aliphatic chains) of lignin was observed at 3351 cm^−1^. A stretching vibration of C–H from the methyl and methylene groups was observed at 2935 cm^−1^. The aromatic ring vibration spectra observed at 1596, 1510, and 1425 cm^−1^ correspond to the phenylpropane monomers^35^. Also, specific bands attributed to the G unit moieties were observed at 1265, 855, and 815 cm^−1^, indicating the presence of softwood lignin^36^. The in plane deformation vibration at 1456 cm^−1^ indicates C-H superimposed on the lignin aromatic skeleton, and the stretching vibration at 1700 cm^−1^ is attributed to the C = O of the carboxyl group^37^.

**Figure 5:**
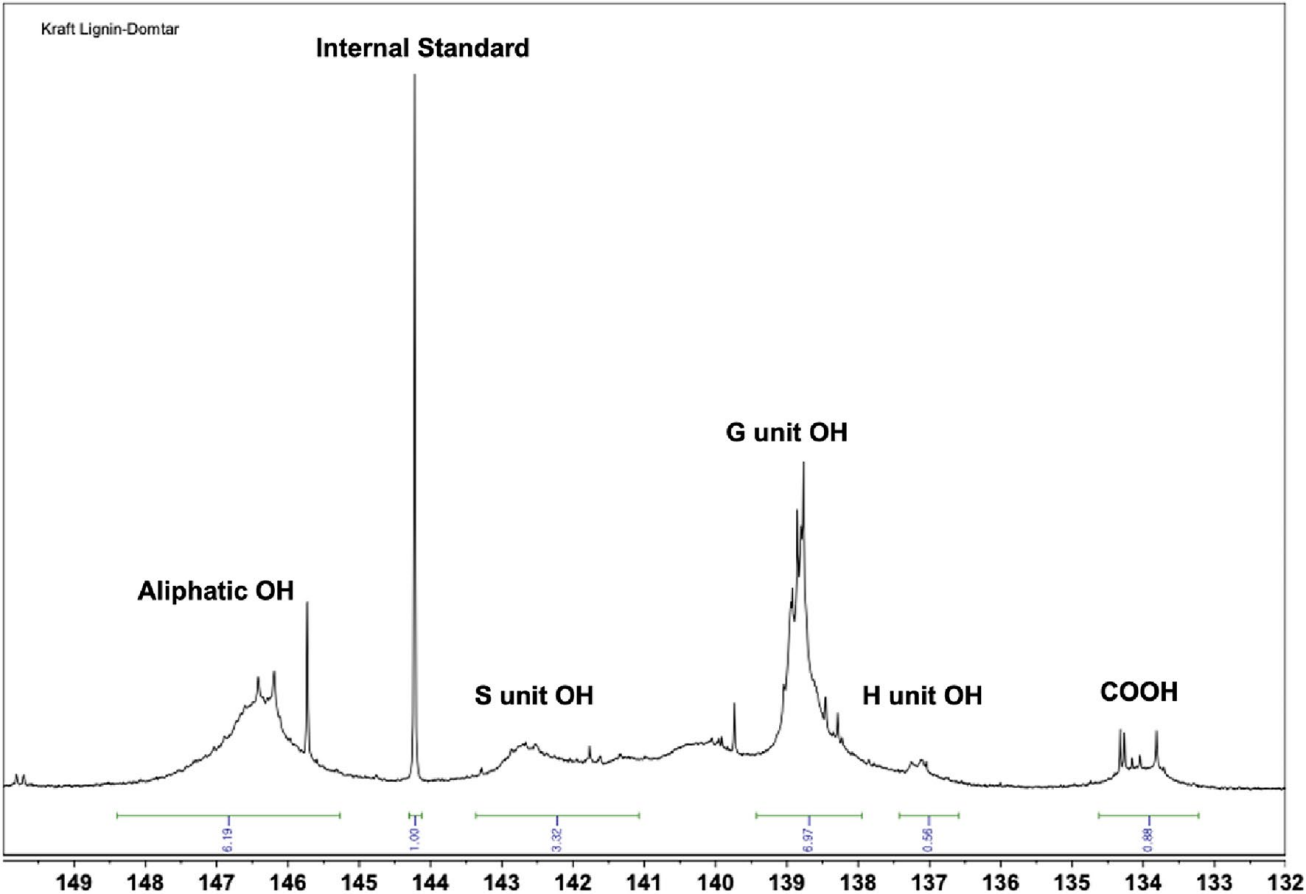
^31^P NMR spectra of soft wood kraft lignin provided by Domtar.

The quantitative data for the hydroxyl groups in lignin gathered through the liquid ^31^P NMR analysis are presented in Table 2. The spectra of ^31^P NMR is presented in Fig. 5. The predominant presence of G units in the lignin indicates that it was from softwood, which aligns with the FT-IR data. The concentration of free hydroxyl groups in KL is 4.9 mmol/g, encompassing three types of functional groups: 62% of phenolic, 33% of aliphatic, and 5% of carboxylic acid. It is the hydroxyl groups in lignin that are expected to function as a bio-based polyol in the PU matrix^38^.

### Hydroxyl numbers, molecular weight distributions, and viscosities of polyols

As presented in Table 3, the hydroxyl number of lignin was calculated to be 328 mg KOH g^−1^. The average molecular weight of the lignin samples was determined to be 4302 g/mol, consistent with typical observations in softwood KL^39^. The number-average molecular weight of lignin was 1483 g/mol, and the polydispersity index was 2.9, indicating structural heterogeneity. Compared with PEG 400, the KL had a significantly larger molecular weight and less uniformity, contributing to lower reactivity in its powdered form. Similarly, the castor oil exhibited a lower hydroxyl number than the PEG 400, which was accompanied by a viscosity that is six times higher and a molecular weight that is two times larger than that of PEG 400. These results suggest that biomass-derived polyols such as KL and castor oil exhibit lower reactivity with isocyanate than a conventional polyol.

**Table 3:** The hydroxyl number, viscosity, and molecular weight distribution of reagents used.

Reagents	Hydroxyl number (mg KOH g^−1^)	Viscosity (Pa·S)	M_W_	M_m_	PDI^a^
PEG 400	293	0.20	503	420	1.2
Lignin	328	–	4302	1483	2.9
Castor oil	163	1.26	915	705	1.3

^a^Polydispersity index (M_w_/M_n_).

### Characterization of lignin-incorporated PU foams

#### Chemical reaction within the PU foam matrix

**Figure 6:**
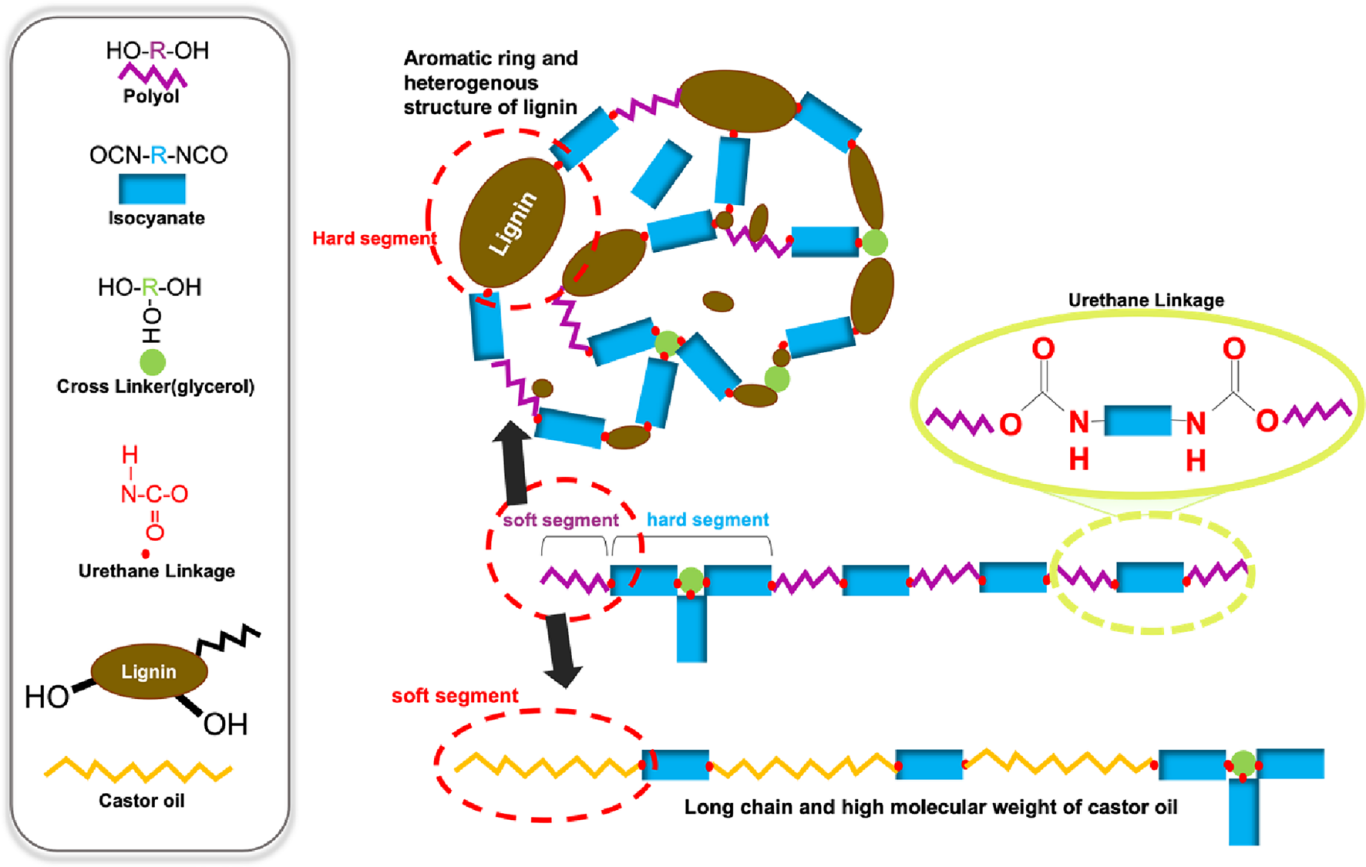
(**a**) Formation structure of lignin-incorporated PU foam; the traditional polyol, which takes part in soft segment int the PU foam matrix, was replaced by lignin known as a hard segment due to its aromatic ring and complex structure. Lignin, with its varying molecular weight distribution, produced polyurethane linkage (bond formation) with isocyanate while being distributed in the matrix, making PU foam matrix more heterogeneous and rigid to some extent, (**b**) Formation structure of castor oil-incorporated PU foam; the traditional polyol was substituted by castor oil, referred to as soft segment. Owing to its long chain structure and higher molecular weight distribution compared to the traditional polyol, the resulting foams are expected to exhibit less rigid structure.

In Fig. 4b,c, The FTIR spectra of the LPU and lpu foams were analyzed to investigate the reactions that produced them. Urethane linkages formed through the polyaddition reaction between hydroxyl groups and isocyanate groups, resulting in the presence of an N–H bond (3300 cm^−1^), C–H stretching of the aliphatic chains (2870 cm^−1^), C=O stretching (1710 cm^−1^), C–N (1218 cm^−1^), and C–O bond (1067 cm^−1^) peaks in all spectra. Carbon–carbon stretching vibrations in the aromatic ring were observed at 1579 cm^−1^ and 1513 cm^−1^, and C–H deformation of the aromatic groups was present in the regions of 815 and 763 cm^−1^^40,41^. The N–H bond at 3300 cm^−1^, characteristic of PU, became broader as the lignin content increased, suggesting that the isocyanate groups successfully reacted with the hydroxyl groups in both the polyol and lignin, and the O–H bond at 3300–3400 cm^−1^ was merged with the N–H bond^42^. The residual isocyanate groups (N=C=O) show absorption at 2275 cm^−1^. In both the LPU and lpu foams, N=C=O increased as the lignin content increased^43^. The gradually widening of the N=C=O bond indicates decreased reactivity for urethane formation in the foams due to the lower reactivity of lignin compared with the conventional polyol^26^. In other words, a broader N=C=O is exhibited with an increase in lignin content due to the lower crosslinking between lignin and isocyanate. Furthermore, compared with LPU, lpu has a higher NCO peak, which is expected to exist in the foams due to the excess amount of isocyanate left after the reaction. The anticipated structure of PU foam following the bond formation between lignin and isocyanate was illustrated in Fig. 6.

#### Mechanical properties and morphology

**Figure 7:**
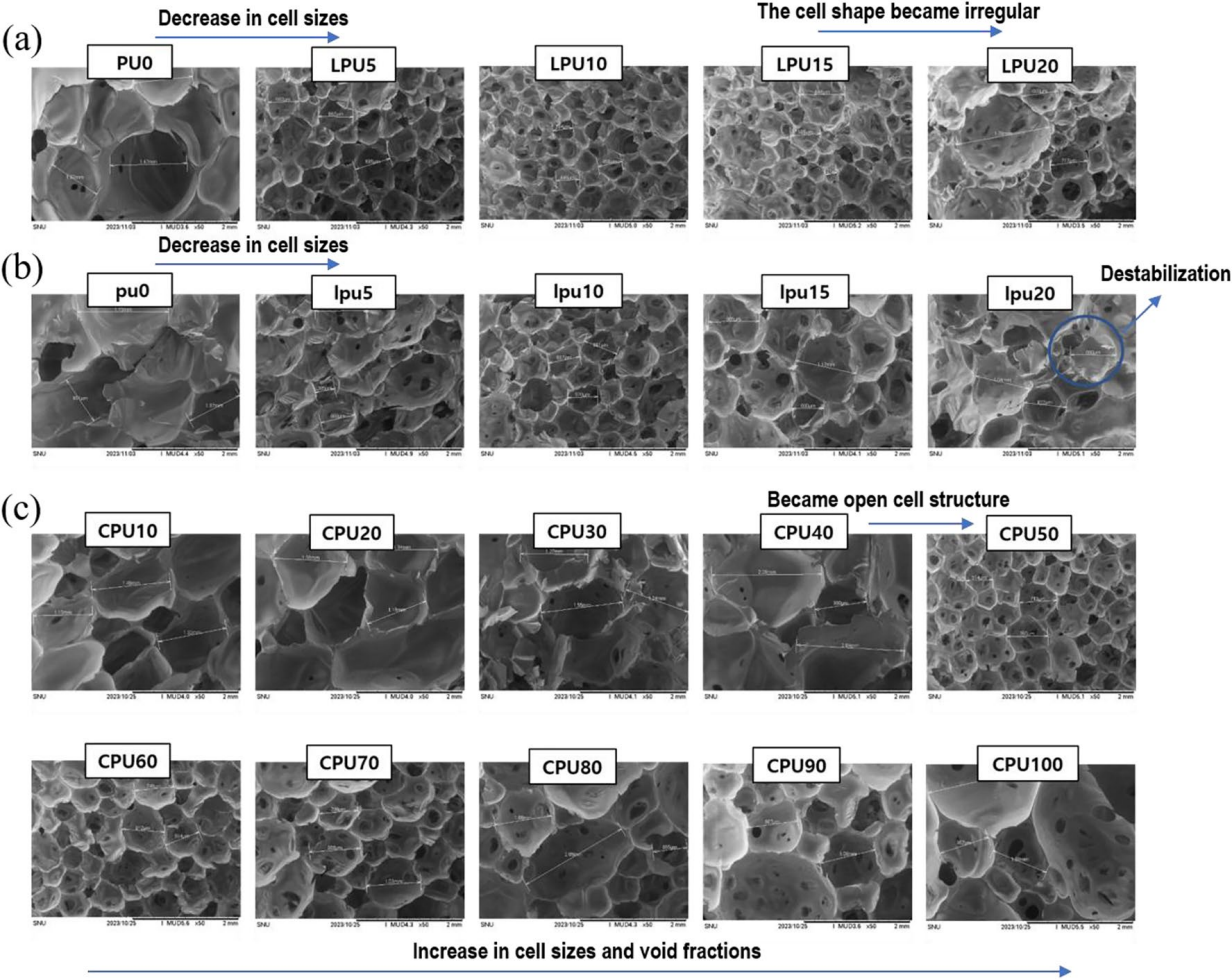
Bio-based polyurethane foam structure obtained by SEM images. (**a**) LPU foams with the addition ~ 20 wt% kraft lignin (index 1.01), (**b**) lpu foams with the addition of ~ 20 wt% of kraft lignin (index of 1.3), (**c**) CPU foams with the addition of ~ 100 wt% of castor oil (index of 1.01).

The addition of lignin as an alternative to the conventional polyol reduced the compressive strength compared with both the PU0 (326 kPa) and pu0 (441 kPa) controls, as demonstrated in Table 4. This decline occurred because the conventional polyol underwent a more rapid chemical reaction with isocyanate, creating a homogeneous and greater crosslinking structure than the lignin-based polyol^38^. At the same time, KL disturbed chemical reaction between polyol and isocyanate, resulting in reduced the number of urethane bonds, which is weaker PU foam matrixes^44^. However, the enhancement in compressive strength from LPU5 (147 kPa) to LPU20 (207 kPa) can be attributed to various factors. First, lignin possesses an aromatic ring structure that contributes to a higher crosslinking density with the NCO groups of the isocyanate and increased chain stiffness^45^. Second, as the lignin content in the bio-based polyol increased, the reactivity with isocyanate is assumed to decrease because it is less uniform and more viscous than a conventional polyol^46^. Consequently, the remaining unreacted isocyanate reacted with another hydrogen bond while forming allophanate groups, leading to enhanced crosslinking density^26^. Third, the cell size decreased slightly as the lignin content increased due to the slow polyaddition reaction rate, leading to the release of more CO_2_ gas during formation of the foam structure, which affected the shape of the cells and cell walls, including the spherical struts and strut joints shown in the SEM images in Fig. 7a,b. Since lignin affects the porosity of the foam as well as distribution, the pore size of the resulting foams was also observed and presented in Table 4. At a 10 wt% lignin loading level, the pore size of both foams are the smallest and most homogeneous. This reduction in pore size has been reported in several studies of lignin-incorporated PU foam^32^.

**Table 4:** Mechanical and thermal properties of bio-based polyurethane foams.

Index	Sample	Compressive strength (KPa)	Apparent density (kg/m3)	Pore size (µm)	TG analysis
		At 10% of compression strain			Char yield (wt%)
1.01	PU0	326 ± 1.9	79.1 ± 1.4	1207 ± 235	10.2
	LPU5	147 ± 11.1	49.7 ± 3.0	646 ± 58	11.5
	LPU10	147 ± 10.9	51.7 ± 3.8	432 ± 33	12.3
	LPU15	168 ± 12.5	56.2 ± 1.6	545 ± 314	14.4
	LPU20	207 ± 3.4	52.5 ± 2.4	1062 ± 622	15.8
1.3	pu0	441 ± 10.1	81.1 ± 3.3	1250 ± 420	13.0
	lpu5	164 ± 4.0	48.0 ± 1.2	681 ± 303	12.4
	lpu10	163 ± 4.6	46.0 ± 0.5	613 ± 46	13.8
	lpu15	167 ± 7.7	46.1 ± 1.3	912 ± 289	15.0
	lpu20	147 ± 8.9	49.0 ± 2.2	907 ± 116	17.5
1.01	CPU10	284 ± 25.2	60.6 ± 6.6	1313 ± 180	10.6
	CPU20	255 ± 15.5	59.1 ± 5.9	1300 ± 106	10.2
	CPU30	237 ± 31.2	59.5 ± 6.0	1353 ± 171	8.4
	CPU40	207 ± 26.9	58.9 ± 7.3	1703 ± 618	6.6
	CPU50	161 ± 38.4	52.1 ± 4.5	669 ± 138	5.9
	CPU60	144 ± 5.4	57.6 ± 3.7	745 ± 64	6.1
	CPU70	116 ± 18.7	60.3 ± 2.9	912 ± 153	5.7
	CPU80	104 ± 5.4	58.5 ± 2.2	1275 ± 737	5.5
	CPU90	84 ± 15.1	60.9 ± 1.8	978 ± 97	4.6
	CPU100	23 ± 0.5	73.2 ± 3.6	1416 ± 690	4.2

The index ratios of LPU and lpu were 1.01 and 1.3, respectively. Thus, lpu exhibited overall higher compressive strength due to its higher isocyanate content, which is the hard segment in a PU matrix^47^. The compressive strengths of the lpu foams were 164 kPa (lpu5), 163 kPa (lpu10), 167 kPa (lpu15), and 147 kPa (lpu20), whereas that of the control pu0 foam was 441 kPa. Among the lpu samples, the compressive strength did not change significantly as the lignin content increased^28^. However, beyond a certain amount of lignin, as observed in lpu20, the foaming process was affected^48^, leading to dimensional destabilization of the PU foam structure, as shown in Fig. 7b. This destabilization occurred because a significant amount of unreacted isocyanate remained, and an excess of the solid powder form of lignin negatively affected the foam system. For these reasons, the cell shape became irregular and pore size became less uniform as the lignin content in the LPU and lpu foams increased from 15 wt% of lignin loading level. The self-association of the lignin polyol is attributed to the adherence of particles to the cell wall, which ultimately results in the distortion or rupture of the cell shape^49^. The apparent density of LPU and lpu foams containing 5 wt% to 20 wt% lignin, did not differ significantly. However, compared with the control foam (79.1 kg/m^3^ and 81.1 kg/m^3^), the apparent density of LPU and lpu foams decreased along with the compressive strength because of the lower crosslinking density, as explained above.

#### Thermal behavior

**Figure 8:**
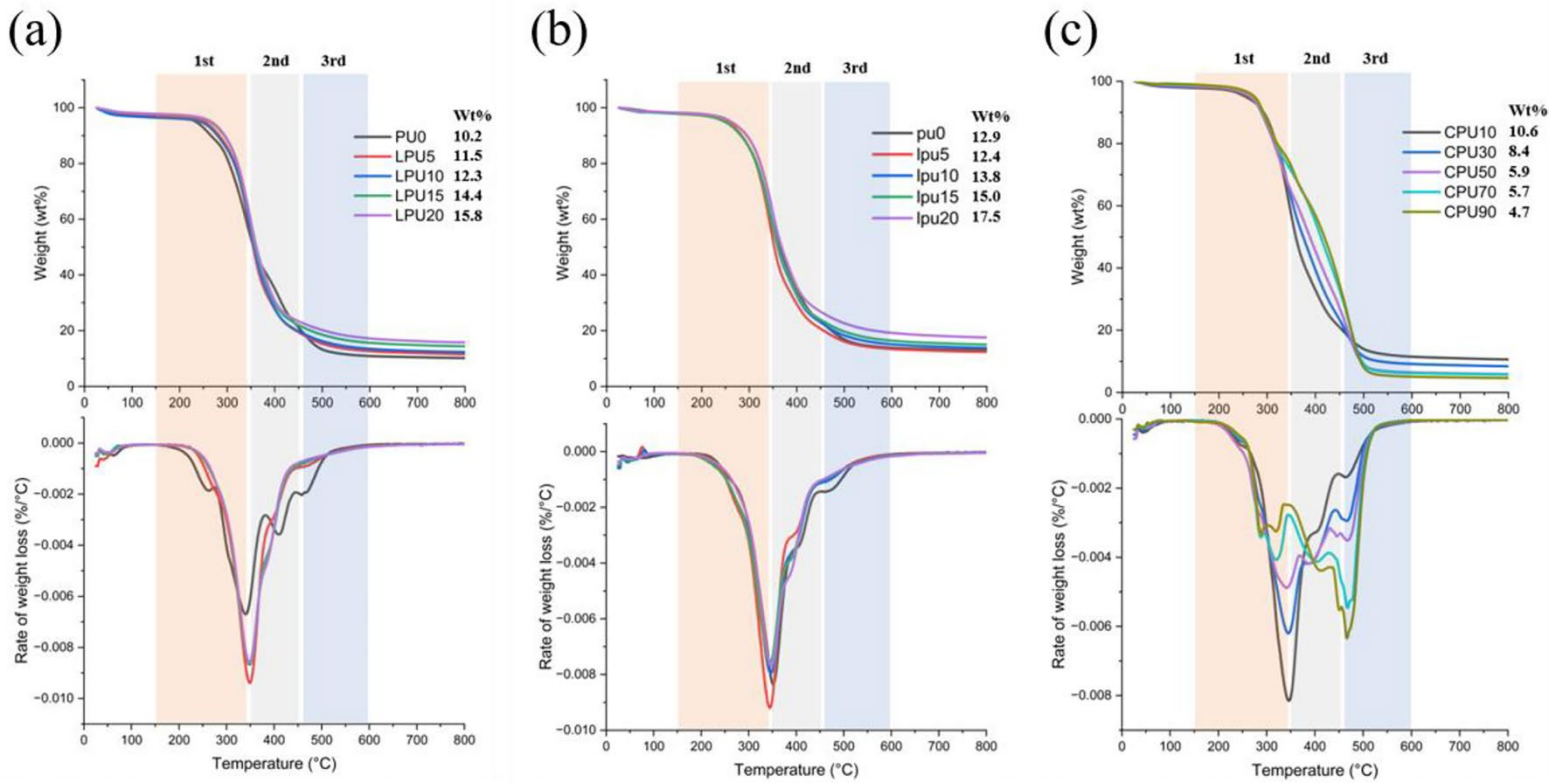
TG and DTG curves of (**a**) LPU, (**b**) lpu and (**c**) CPU foams.

Figure 8 depicts the TG and differential thermogravimetric (DTG) curves for both the LPU(a) and lpu foams(b). The char yield increased gradually from 10.2 wt% for PU0 to 15.8 wt% for LPU20 and from 12.9 wt% for pu0 to 17.5 wt% for lpu20. The char yield suggests that increasing the amount of lignin in both the LPU and lpu foams produced higher thermal stability. This behavior was attributed to the thermostability of lignin^50^. In detail, the foams exhibited stability below 150 °C, followed by three major thermal decomposition patterns. The initial event at 150–350 °C was attributed to the removal of the hard segment and the cleavage of urethane bonds^25^. The second stage, from 350 to 450 °C, was associated with weight loss from the soft segment in the foams. The third phase occurred at 450–600 °C and indicated the breakdown of the lignin aromatic ring network. Due to the intrinsic structure of lignin, in the third stage, the thermal degradation of lignin-containing foams is generally less than that of control foams^40^. All foam samples underwent degradation within the temperature range of 150–600 °C, with the peak degradation occurring in the range of 320–350 °C. In this range, the control foam had the minimum degradation rate because it had the highest crosslinking of urethane bonds, as shown by the FTIR spectra and compression strength^51^. At the same time, an increase in lignin content corresponded to a decrease in the trend of the maximum degradation rate because the intrinsic and aromatic structure of lignin enhanced the stiffness of the polymer chains, leading to a low degradation rate^52^.

### Characterization of castor oil-incorporated PU foams

#### Chemical reaction within the PU foam matrix

Figure 4d shows the infrared spectra results for PU0, CPU50, and CPU100. Similar to the LPU and lpu foams, a peak in the range of 3333–3294 cm^−1^, corresponding to the N–H bond, was observed, indicating the successful urethane reaction in the foam matrix. The FT-IR peaks at 2925 cm^−1^ and 2854 cm^−1^ confirmed the presence of the C–H stretching vibration^53^. As the castor oil content increased, the C-H spectrum widened. Specifically, the CPU foams containing 0 wt% to 100 wt% of castor oil underwent successful reactions that resulted in the absence of remaining isocyanate (N=C=O) and stretching vibrations of the O–H groups. In other words, all of the isocyanate reacted with all the polyols, leading to urethane linkages^54^. Moreover, as the amount of castor oil increased, a broader spectrum of stretching vibrations indicating C=O bonds was observed in the region of 1720 cm^−1^ due to the structure of the castor oil and not only from the urethane linkages^55^. The stretching vibration at 1216 cm^−1^ can be associated with C–N bonds in urethane linkages^53^. Following section will be explained about possible bond formation between castor oil and isocyanate as described in Fig. 6.

#### Mechanical properties and morphology

In the CPU foams, the compressive strength decreased gradually as the amount of castor oil increased. As shown in Table 4, CPU10 exhibited a compressive strength of 284 kPa, and CPU100 showed a reduced strength of 23 kPa. This decrease with higher castor oil content is due to lower crosslinking and a reduced number of hard segments because castor oil has a long chain structure, as demonstrated in Figs. 1 and 8. Therefore, it acts as a soft segment, leading to the formation of semi-rigid foams^45^. In other words, the polymer chains get less tightly packed together as the amount of castor oil increases, and cell morphology affects the mechanical properties. The increase in castor oil content caused larger cell sizes and void fractions, consistent with the SEM images in Fig. 7c. Notably, when the castor oil content reached 50 wt%, the foam exhibited an open cell structure, which also indicates that the soft segments increase along with the higher castor oil content. The transition from a closed-cell to an open-cell structure occurred at a castor oil content of 50 wt%. At this content, the pore size decreased to 669 µm compared to 1313–1353 µm observed at 10–40 wt% content, as shown in Table 4. However, the pore size increased again from 669 µm (CPU50), reaching 1416 µm (CPU100). These changes in cell structure and pore size were accompanied by a decrease in compressive strength^33^. Although the compressive strength of the CPU foams decreased as the castor oil content increased, the apparent density within the CPU foams was similar to that of the control^56^.

#### Thermal stability

Figure 8c shows the TG and DTG curves for the CPU foams. As the amount of castor oil in the foams increased, lower thermal stability and a reduced char yield were observed. The char yield decreased gradually from 10.6 wt% with CPU10 to 4.2 wt% for CPU100 (Table 4). More specifically, three distinct stages were observed during the thermal decomposition of the CPU foams. The beginning weight loss stage from 150 °C was caused by the depolymerization of some hard segments within the urethane bonds. At 150–350 °C, foams with higher castor oil content had a lower degradation temperature and faster degradation rate because of the lower hard segment content^57^. The second weight loss stage at 350–450 °C is mainly attributed to the chain scission of the polyols, which is the degradation of the castor oil fatty acid chains^58^. In this stage, the rate of weight loss in CPU foams increased with the addition of castor oil, which could be explained by the increased proportion of soft segments as the castor oil content increased from 10 to 100%. After the decomposition of the urethane bonds and the polyols mentioned above, the final stage is mainly assigned to further thermo-oxidation.

## Conclusion

This study reports a facile and promising method for producing PU foams using lignin and castor oil as bio-based polyols. Due to the presence of hydroxyl groups in their structures, lignin was incorporated up to 20 wt% and castor oil up to 100 wt% by simple mechanical mixing in a one-pot process. This mixture was then blended with other reagents for foam production. Lignin, being a less reactive chemical with a complex structure, influenced the foaming process by producing more homogeneous and smaller pore sizes within the PU foam to some extent. However, incorporating beyond certain amounts of lignin caused destabilization due to unreacted isocyanate and excess of solid powder, which affected the foaming process. Additionally, thermal stability improved with increasing lignin content, resulting in higher char residue due to the aromatic structure of lignin. Thus, optimal amount of lignin can be expected to enhance thermal stability in rigid foams. On the other hands, castor oil incorporation suggests that it can be used for semi-rigid foams with less packed structure and reduced thermal stability due to its long chain structure. In conclusion, this study demonstrated the effects of lignin and castor oil as bio-based polyols in PU foam synthesis and highlighted their potential value depending on the desired application. This research serves as a foundational study to contribute to further bio-based PU foam research.

## Data Availability

The datasets used and/or analyzed during the current study available from the corresponding author on reasonable request.
